# Resting-State Network Disruption and *APOE* Genotype in Alzheimer's Disease: A *lagged* Functional Connectivity Study

**DOI:** 10.1371/journal.pone.0046289

**Published:** 2012-09-25

**Authors:** Leonides Canuet, Ivan Tellado, Veronica Couceiro, Carmen Fraile, Lucia Fernandez-Novoa, Ryouhei Ishii, Masatoshi Takeda, Ramon Cacabelos

**Affiliations:** 1 EuroEspes Biomedical Research Center, Institute for CNS Disorders and Genomic Medicine, Corunna, Spain; 2 EuroEspes Biotechnology Division (Ebiotec), Institute for CNS Disorders and Genomic Medicine, Corunna, Spain; 3 Department of Psychiatry, Osaka University Graduate School of Medicine, Suita city, Osaka, Japan; University Medical Center Groningen UMCG, The Netherlands

## Abstract

**Background:**

The apolipoprotein E epsilon 4 (*APOE*-4) is associated with a genetic vulnerability to Alzheimer's disease (AD) and with AD-related abnormalities in cortical rhythms. However, it is unclear whether *APOE-*4 is linked to a specific pattern of intrinsic functional disintegration of the brain after the development of the disease or during its different stages. This study aimed at identifying spatial patterns and effects of *APOE* genotype on resting-state oscillations and functional connectivity in patients with AD, using a physiological connectivity index called “*lagged* phase synchronization”.

**Methodology/Principal Findings:**

Resting EEG was recorded during awake, eyes-closed state in 125 patients with AD and 60 elderly controls. Source current density and functional connectivity were determined using eLORETA. Patients with AD exhibited reduced parieto-occipital alpha oscillations compared with controls, and those carrying the *APOE*-4 allele had reduced alpha activity in the left inferior parietal and temporo-occipital cortex relative to noncarriers. There was a decreased alpha2 connectivity pattern in AD, involving the left temporal and bilateral parietal cortex. Several brain regions exhibited increased lagged phase synchronization in low frequencies, specifically in the theta band, across and within hemispheres, where temporal lobe connections were particularly compromised. Areas with abnormal theta connectivity correlated with cognitive scores. In patients with early AD, we found an *APOE*-4-related decrease in interhemispheric alpha connectivity in frontal and parieto-temporal regions.

**Conclusions/Significance:**

In addition to regional cortical dysfunction, as indicated by abnormal alpha oscillations, there are patterns of functional network disruption affecting theta and alpha bands in AD that associate with the level of cognitive disturbance or with the *APOE* genotype. These functional patterns of nonlinear connectivity may potentially represent neurophysiological or phenotypic markers of AD, and aid in early detection of the disorder.

## Introduction

Alzheimer's disease (AD) is characterized by a marked desarborization of synaptic contacts and neuronal loss, leading to progressive memory deficits and cognitive decline [1]. Pathological changes in the brain, namely intracellular aggregates of tau protein filaments (neurofibrillary tangles) and extracellular deposition of beta amyloid peptides (amyloid plaques), constitute the hallmarks of the disease [2]. The search for biomarkers or early biological signs of AD has revealed genetic alterations as important risk factors for developing the disease, in particular the presence of the epsilon 4 allele of the apolipoprotein E (*APOE*-4) gene [3]–[7]. In addition, neuroimaging studies have provided substantial evidence indicating that atrophy and dysfunction in brain regions subserving cognitive function are closely associated with pathological aging or with the presence of the *APOE-*4 allele [8], [9].

Given that characterizing brain activity purely in terms of anatomically segregated responses is not sufficient to explain the complexity of AD symptomatology, recent studies have focused their attention on functional connectivity between brain regions using functional Magnetic Resonance Imaging (fMRI) [10]–[16], electroencephalography (EEG) [17]–[19] or magnetoencephalography (MEG) [20]–[22]. Functional connectivity refers to the temporal synchrony or correlation between signals of two or more spatially separated regions as an index of functional integration between neural populations [23]–[25]. Findings from most neuroimaging and neurophysiological studies proposed that disrupted functional connectivity represents a core pathophysiological mechanism underlying AD. This is supported by reports of white matter structural abnormalities in AD, using diffusion tensor imaging or MRI tractography, as measures of anatomical connectivity [26]–[28]. This evidence highlights the importance of regarding AD as a functional and structural network disorder.

The *APOE-*4, a major genetic risk factor for sporadic AD, has been found to affect gray matter and white matter structure as well as cortical rhythms and functional/structural connectivity in healthy subjects long before the onset of clinical dementia [29]–[32]. Likewise, subjects carrying the *APOE*-4 allele who already developed AD also have abnormal patterns of cortical oscillations compared with noncarriers [33], [34]. However, limited work has suggested that *APOE* genotype may modulate disease phenotype, in terms of brain functional connectivity [21], [35], and it is unclear whether the effects of *APOE*-4 on brain connectivity change throughout the stages of the disorder.

For a better understanding of AD pathophysiology, functional connectivity is commonly assessed during performance of a cognitive task. However, recently, special interest has been paid to the intrinsic functional organization of brain networks in resting state [12], [14], [16], [36]. The brain resting state is thought to be an energetically costly condition characterized by a rich neural activity and long-range interneuron connections in specific brain circuits (e.g., the default mode network or DMN) that are temporally interrupted or attenuated during performance of sensorimotor or cognitively-demanding tasks [37]. This intrinsic functional organization during rest allows the brain to allocate resources and ready itself for changes or stimuli in internal and external environments. Therefore, the investigation of functional connectivity during resting state rather than during a particular task may reveal an intrinsic functional disintegration between brain regions in AD, and its association with genetic risk factors for this disorder.

To visualize resting-state synchronization across frequency bands in large-scale functional networks, various methods of connectivity between pairs of electrodes have been applied to EEG recordings. Most studies of AD connectivity assessed coherence, a linear connectivity measure that depends on the magnitude or power of the signals, and suggested reduced connections in different brain networks [17], [38], [39]. A few studies explored EEG nonlinear connectivity using pair-wise electrode functional coupling or synchronization likelihood measures [12], [21], [22]. Nonlinear measures potentially offer higher sensitivity due to the generality of the dependence structures they are able to capture [40]. However, it is well known that EEG suffers from the problem of volume conduction or common sources, which gives rise to spurious correlations between time series recorded from neighboring electrodes [41]. Thus, in sensor-level connectivity, activity of an underlying source is detected instantaneously (zero-lag) by different scalp electrodes. This field spread severely limits the utility of connectivity measures computed directly between sensor recordings. Exact Low Resolution Electromagnetic Tomography (eLORETA) represents a feasible solution to this problem as it assesses neuronal interactions at the level of the reconstructed sources by computing cortical current density and dynamic functional connectivity based on the estimated current density signals with optimal localization [24].

A recently developed measure of nonlinear functional connectivity called “*lagged phase synchronization*”, implemented in the eLORETA statistical package, is resistant to non-physiological artifacts, in particular low spatial resolution and volume conduction, that usually affect previously published connectivity indices (e.g., phase coherence, imaginary component of coherence, classic phase synchronization) [24], [41], [42]. This lagged connectivity measure is thought to be accurately corrected as it represents the connectivity between two signals after the artifactual instantaneous zero-lag contribution has been excluded. For instance, the connectivity patterns of the classic phase synchronization (which include the instantaneous artifact) are dominated by the locations of maximum activity, which are seen as common instantaneous sources, and therefore are not often related to true physiological connections [24]. Compared to the imaginary part of the coherence proposed by Nolte et al. [42] as a conservative index of connectivity, the lagged connectivity measure has the important property of being relatively robust to the strength of the instantaneous component, i.e. it can still detect physiological “non-zero” lagged connectivity even in the presence of large instantaneous artifacts, while the imaginary part of the coherence fails to detect a lagged connection by tending to zero if the instantaneous component is large [24]. This would also apply to phase synchronization, since it is nothing more than the coherence for unit modulus (amplitude-free) Fourier transform coefficients. The phase lag index proposed by Stam et al. [41] represents an improvement over the imaginary part of the coherence because it is less affected by phase delay. However, it is also relatively insensitive to true changes in phase synchronization when the phase lies very close to zero, which is likely to happen as the instantaneous component increases. Because of a proper model for the two components of a connection (i.e., instantaneous and lagged), eLORETA connectivity algorithm is thought to detect genuine physiological connectivity. Furthermore, it can be applied to filtered data, thus giving a frequency decomposition of brain connectivity [24].

The purpose of this study is to identify abnormal EEG patterns and the effects of *APOE*-4 on resting-state oscillations and intrinsic functional connectivity, using the lagged phase synchronization index, and to assess correlations between connectivity patterns and the cognitive level. We also aimed at determining whether the effects of *APOE*-4, as a susceptibility factor, are seen in different stages of AD severity.

## Methods

### Subjects

The study was conducted in 125 patients with probable AD who attended the outpatient clinic at the EuroEspes Biomedical Research Center from January 2004 to June 2011. We also recruited 60 elderly subjects who acted as healthy controls (HC). They consisted of neurologically and psychiatrically normal subjects visiting our center for clinical check-up or genetic and pharmacogenomic screening. All patients met the diagnosis of probable AD according to the National Institute of Neurological and Communicative Disorders and Stroke/the Alzheimer's Disease and Related Disorders Association (NINCDS-ADRDA) [43] and the DSM-IV [44] criteria. They underwent standard screening which involved physical and neuropsychiatric examination, laboratory tests (e.g., serum cholesterol, folic acid, vit B_12_, HIV, thyroid hormones), a neuropsychological battery, quantitative electroencephalogram (EEG), and neuroimaging tests using MRI or Computed Tomography (CT). The neuropsychological battery included the Alzheimer's Disease Assessment Scale-cognitive subscale (ADAS-cog) as a global rating scale that measures cognitive symptoms of the disease. The severity of dementia was assessed with the Mini-Mental State Examination (MMSE) [45] in all patients. In addition, the Global Deterioration Scale (GDS) was also assessed in most of the patients (n = 118). Accordingly, the patients were divided into three severity groups: early or mild AD (MMSE score: 19–24, and GDS score: 3 or 4), moderate AD (MMSE score: 10–18, or GDS score: 5), and severe AD (MMSE score: ≤9, or GDS score: 6) group [16], [45], [46]. All these tests allowed us to rule out brain structural lesions as well as different causes of progressive or reversible dementias. Patients with a history of brain trauma or drug/alcohol abuse were not included. Out of 232 patients with a diagnosis of dementia that were initially recruited, 107 were excluded for having brain structural lesions, complications of a systemic disorder (e.g., renal failure, diabetes), or non-AD dementia. *APOE* genotyping was also performed on all participants. Like the patients, controls also underwent physical and neuropsychiatric examination, neuropsychological tests, and neuroimaging screening. All the tests in patients and controls were part of routine screenings or check-ups that took place prior to the start of the study. Table 1 shows the demographic and clinical (i.e., cognitive scores) characteristics of the participants. None of them was taking antipsychotics, sedatives, antidepressants or cholinesterase inhibitors at the time of recruitment. Prior to the beginning of the study, all the procedures were explained in detail to the participants by qualified neuropsychiatrists, including the authors of this study (RC and LC). Subsequently, written informed consent was obtained from all capable participants. In those considered to have reduced capacity to understand the informed consent document due to their cognitive deficits, a legal representative or caregiver consented on their behalf. This study and the consent procedures were approved by the institutional review board of the EuroEspes Medical Center, in line with the ethical code of the World Medical Association (Declaration of Helsinki).

**Table 1:** Demographic and clinical characteristics.

Characteristics	Alzheimer's disease				Controls	P-value^a^
	Early *n* = 38	Moderate *n* = 70	Severe *n* = 17	Total *n* = 125	*n* = 60	
Age (*mean±SD*)	70.9±5.9	72.7±8.1	70.6±9.5	71.9±7.7	67.3±6.8	0.19
Gender (*F/M*)	22/16	48/22	12/5	82/43	24/36	0.001
MMSE (*mean±SD*)	21.8±1.6	13.6±2.9	5.9±2.7	15.2±5.7	28.8±0.9	0.000
ADAS-cog (*mean±SD*)	20.1±7.9	35.9±11.4	46.8±8.1	31.6±13.1	7.9±3.5	0.000
*APOE ε*4 carrier (*n*)	17	34	9	60	12	0.000
*APOE ε*4 noncarrier (*n*)	21	36	8	65	48	0.000

Data are mean ± SD unless otherwise noted.

a Comparison between the total sample of patients and controls. MMSE, Mini-mental State Examination; ADAS-cog, Alzheimer's Disease Assessment Scale-cognitive subscale; *APOE*-4, apolipoprotein E epsilon 4.

### APOE genotyping

*APOE* genotype was determined after DNA extraction from peripheral blood samples using the standard polymerase chain reaction (PCR) method [4]. The subjects were classified into two groups according to their *APOE* status: epsilon 4 allele carriers (subjects having at least one allele) and noncarriers.

### EEG Recordings and Data Acquisition

Digital 19-channel scalp EEG (Neuroscan Medical Systems, Neurosoft Inc. Sterling, VA, USA) was recorded during awake-resting condition, with the electrodes fixed in an elastic cap (ECI-Electro cap, Eaton, Ohio, USA), and positioned according to the International 10–20 system (i.e., Fp1, Fp2, F7, F3, Fz, F4, F8, T7, C3, Cz, C4, T8, P7, P3, Pz, P4, P8, O1, O2). The EEG activity was acquired using a linked ears reference, sampled at 250 Hz, and filtered offline between 1 and 30 Hz. Electrode impedance was kept below 5 kΩ. Vigilance-controlled EEG recordings lasted about 5 to 6 minutes, including eyes closed and eyes open states, as well as photic stimulation in all subjects. Analyses were circumscribed to the 3 min of awake, eyes-closed condition. For each subject, 40 s of artifact-free EEG data, fragmented into epochs of 2 s, were randomly selected. Artifact rejection was performed manually based on visual inspection by an experimenter blind to the participants' condition and genetic data. Segments containing blinking artifacts, muscle or cardiac contamination, or drowsiness signs were carefully excluded, so that reliable estimates of brain function in the awake, resting-state could be obtained. Further analyses were performed using the LORETA-KEY software package as provided at www.uzh.ch/keyinst/LORETA.html.

### EEG-Source Localization Analysis

To compute the intracortical distribution of the electric activity from the surface EEG data, we used eLORETA [24]. The eLORETA method is a discrete, three-dimensional distributed, linear, weighted minimum norm inverse solution. The particular weights used in eLORETA endow the tomography with the property of exact localization to test point sources, yielding images of current density with exact localization albeit with low spatial resolution (i.e., neighboring neuronal sources will be highly correlated). A further property of eLORETA is that it has no localization bias even in the presence of structured noise. In this sense, eLORETA is an improvement over previously related tomographies LORETA [47] and the standardized version sLORETA [48].

The current eLORETA implementation employs a three-shell spherical head model and EEG electrode coordinates derived from cross-registrations between spherical and realistic Talaraich head geometry. The head model and the electrode coordinates were registered to the Montreal Neurologic Institute average MRI brain (MNI152) [49]. The solution space was restricted to the cortical gray matter, corresponding to 6239 voxels at 5×5×5 mm spatial resolution. Validation for the eLORETA tomography rests upon the abundant published validation for the previous LORETA and sLORETA methods using fMRI [50], [51], structural MRI [52], PET [53], and intracranial EEG recordings [54].

Selected artifact-free EEG segments were used for calculating the eLORETA intracranial spectral density with a resolution of 1 Hz, from 1 to 30 Hz. eLORETA functional images of spectral density were computed for six frequency bands: delta (1.5–4 Hz), theta (4–8 Hz), alpha1 (8–10 Hz), alpha2 (10–13 Hz), beta1 (13–20 Hz) and beta2 (21–30 Hz).

### Functional Connectivity Analysis

For functional connectivity analysis, we applied a voxel-wise approach, using the electrode sites to define regions of interest (ROI). To create the ROIs, eLORETA determined the MNI coordinates for the cortical voxels underlying the 19 electrodes of the 10–20 system [55]. The ROIs are centered at the given voxel coordinates (ROI centroid), and include all cortical gray matter voxels within 15 mm distance from the center [24], [55]. The 19 cortical ROIs determined by eLORETA are shown in Table 2. A recently developed index of physiological connectivity, namely lagged phase synchronization was used as measure of functional connectivity between all pairs of ROIs [23], [24], [55], [56]. Lagged phase synchronization measures the similarity (a corrected phase synchrony value) between signals in the frequency domain based on normalized (unit module) Fourier transforms; thus it is related to nonlinear functional connectivity. This lagged connectivity measure is thought to be accurately corrected as it represents the connectivity between two signals after the instantaneous zero-lag contribution (artifactual component) has been excluded. Such a correction is necessary when using scalp EEG signals or estimated intracranial signals (EEG tomography) because zero-lag connectivity in a given frequency band is often due to non-physiological effects or intrinsic physics artifacts, in particular volume conduction and low spatial resolution that usually affect other connectivity indices [41], [42]. Thus, this measure is thought to contain only physiological connectivity information. Details on eLORETA lagged connectivity algorithm can be found in recent publications [23], [24].

**Table 2:** Cortical regions of interest (ROIs) determined by eLORETA.

Scalp electrodes	ROI centroid MNI coordinates^a^			Anatomical regions	BAs
	*x*	*y*	*z*		
Fp1	−25	65	−5	Left frontopolar cortex	10,11
Fp2	25	65	−5	Right frontopolar cortex	10,11
F7	−50	40	−10	Left IFG/ATC	45,47,38
F3	−45	40	30	Left MFG (DLPFC)	9,46
Fz	5	45	50	Superior frontal gyrus (mPFC)	8,9
F4	45	40	30	Right MFG (DLPFC)	9,46
F8	50	40	−10	Right IFG/ATC	45,47,38
T7/T3	−65	−15	−15	Left STG/MTG	21,22,42
C3	−50	−20	60	Left precentral/postcentral (CC)	1,2,3,4,6
Cz	5	−10	70	Medial frontal cortex	4,6
C4	55	−20	55	Right precentral/postcentral (CC)	1,2,3,4,6
T8/T4	70	−20	−10	Right STG/MTG	21,22,42
P7/T5	−60	−65	−5	Left posterior temporal cortex (TOJ)	19,22,37
P3	−40	−70	45	Left inferior parietal lobe	7,39,40
Pz	−5	−65	65	Medial parietal (Precuneus)	7
P4	45	−70	45	Right inferior parietal lobe	7,39,40
P8/T6	55	−70	0	Right posterior temporal cortex (TOJ)	19,22,37
O1	−20	−100	10	Left occipital cortex	17,18,19
O2	20	−100	5	Right occipital cortex	17,18,19

a ROIs included all cortical voxels within a 15 mm radius of the center. BAs, Brodmann areas; IFG, inferior frontal gyrus; ATC, anterior temporal cortex; MFG, middle frontal gyrus; DLPFC, dorsolateral prefrontal cortex; mPFC, medial prefrontal cortex; STG, superior temporal gyrus; MTG, middle temporal gyrus; CC, central cortex; TOJ, temporo-occipital junction.

### Statistical Analyses

For statistical neuroimaging analysis of source current density, eLORETA applied a statistical nonparametric mapping (SnPM) method [57]. First, the difference in source localization of cortical oscillations between groups for each frequency band was assessed by voxel-by-voxel independent sample *F-ratio*-tests, based on eLORETA log-transformed current density power. In the resulting statistical three-dimensional images, cortical voxels showing significant differences were identified by a nonparametric randomization/permutation procedure (i.e., based on the Fisher's permutation method, thresholded at the 5% probability level) that compares the mean source power of each voxel and the distribution of the permuted values. By estimating the empirical probability distribution for the “maximal-statistic” under the null hypothesis, randomization and permutation tests have proven to be effective in controlling the family-wise error rate (Type I error) in neuroimaging studies [58]. eLORETA used 5000 data randomizations to determine the critical probability threshold values for the actually observed *log F*-ratio values with correction for multiple comparisons across all voxels and all frequencies, without the need to rely on Gaussianity. The use of SnPM applied to LORETA images has been validated in several studies [59], [60]. Details on the nonparametric randomization procedure are provided in the study of Nichols and Holmes [58].

To assess the difference in lagged phase synchronization between pairs of ROIs for each frequency band across groups (AD vs. Controls, and *APOE*-4 carriers vs. noncarriers), eLORETA performed independent sample *t*-tests, thus obtaining *t*-statistic images of brain connectivity. For each analysis, 1026 tests were performed by eLORETA to compare all connections between 19 ROIs (171 connections) for each of the six frequency bands (171×6 = 1026). Furthermore, to correct for multiple comparisons, the eLORETA non-parametric randomization procedure based on “maximal statistic” was used [57], [58]. The omnibus null hypothesis was rejected if at least one *t* value (i.e., voxel t*_max_*) was above the critical threshold t*_crit_* for p = 0.05 determined by 5000 data randomizations. The eLORETA neuroimaging results of source-current density and functional connectivity are not adjusted for demographic data.

To assess the correlation between connectivity measures (i.e., lagged phase synchronization values) and the cognitive level, as an external clinical variable, the product-moment correlations of lagged phase synchronization between all pairs of ROIs and MMSE scores were computed by eLORETA, and the critical probability threshold (p = 0.05) for the data *r*-values, adjusted for multiple comparisons, was determined by the nonparametric randomization procedure [58].

Chi square and *t*-tests for analysis of demographic (i.e., age and sex) and clinical (e.g., neuropsychological tests scores) data were carried out using SPSS software version 16 (SPSS Inc. Chicago, IL).

## Results

### Demographic and Clinical Characteristics

The demographic and clinical characteristics of our sample are given in Table 1. Although there was a female predominance among patients with AD, there was no significant difference in age (p = 0.19) between groups. There was a greater proportion of *APOE*-4 carriers in the patient group (n = 60/48%) compared with controls (n = 12/20%), and the opposite was true for *APOE*-4 noncarriers (AD: n = 65/52%; HC: n = 48/80%). Neuropsychological assessment revealed that patients with AD had significantly lower MMSE scores than controls (p<0.001). The group of early or mild AD was made up by 38 patients, while 70 and 17 patients were included in the moderate and severe group, respectively.

### Source Localization

**Figure 1:**
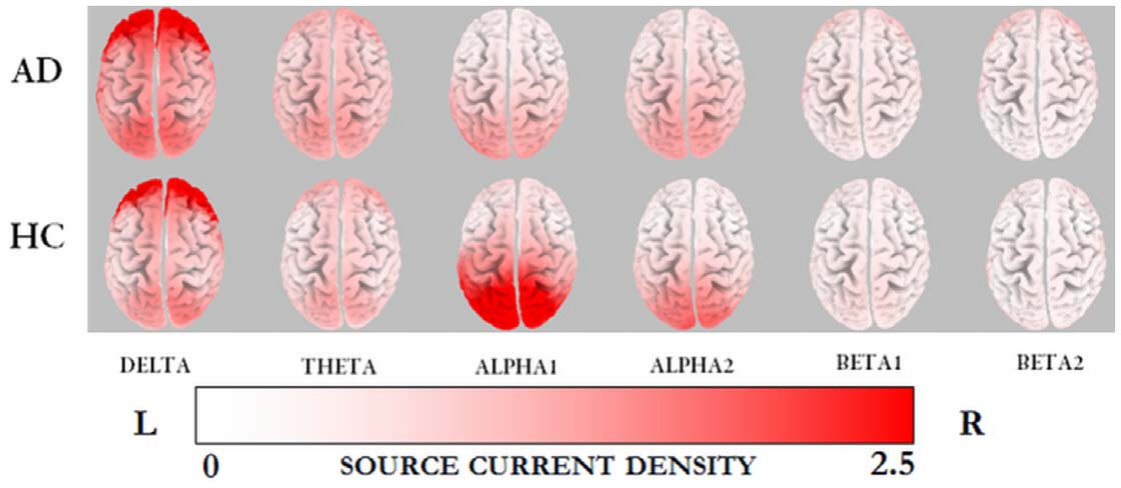
Averaged eLORETA solutions (current density at cortical voxels) of EEG sources for each frequency band in patients and controls. AD, Alzheimer's disease; HC, healthy controls; L, left; R, right.

**Figure 2:**
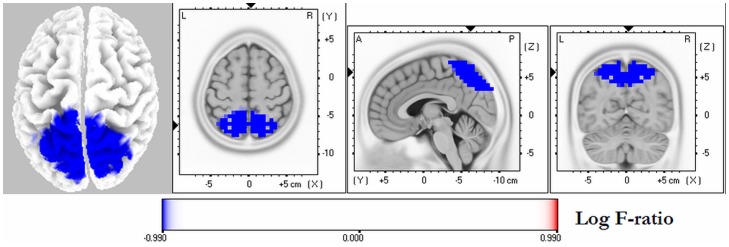
eLORETA statistical maps of alpha1 oscillations in patients with Alzheimer's disease vs. controls. Colored areas represent the spatial extent of voxels with significant difference in source current density (p<0.05, corrected). The MRI slices are located at the MNI-space coordinates of the voxel with highest significance. The color scale represents log F-ratio values (threshold: log F = −0.99, p<0.05). L, left; R, right; A, anterior; P, posterior.

**Figure 3:**
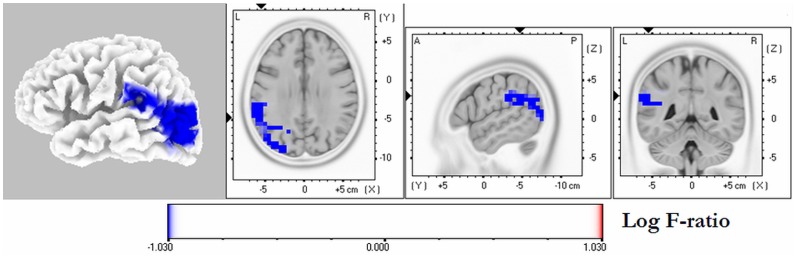
eLORETA statistical maps of alpha1 oscillations in patients with Alzheimer's disease carrying the *APOE*-4 allele vs. noncarriers. Colored areas represent the spatial extent of voxels with significant difference in source current density (p<0.05, corrected). The MRI slices are located at the MNI-space coordinates of the voxel with highest significance. The color scale represents log F-ratio values (threshold: log-F = −1.03, p<0.05). L, left; R, right; A, anterior; P, posterior.

The averaged eLORETA solutions in patients and controls for each frequency band are shown in Fig. 1. The highest current density values were found in the delta frequency band (AD 3.5±2.4; HC: 2.6±1.9), followed by alpha1 band (AD: 1.3±0.6; HC: 2.5±1.7). There was a similar cortical distribution of maximal delta activity across groups over the frontal cortex, whereas alpha1 sources with high current density maxima over the parieto-occipital region were seen only in controls. The statistical analysis revealed significant differences between groups exclusively in the alpha1 frequency band, patients with AD exhibiting reduced activity in the parieto-occipital cortex bilaterally, with the right precuneus showing the highest significance (*x* = 10, *y* = −75, *z* = 50; Log *F*-ratio: −1.03, p = 0.034 corrected) (Fig. 2). An increase in delta oscillations in fronto-central regions nearly reached statistical significance (Log *F*-ratio: −0.78, p = 0.063 corrected). Among patients with AD, those carrying the *APOE-*4 allele exhibited a significant reduction in alpha1 activity compared with noncarriers. These oscillations localized to the left temporo-occipital region and the inferior parietal cortex; the latter showing the highest difference (*x* = −64, *y* = −37, *z* = 30; Log *F*-ratio: −1.05, p = 0.045 corrected) (Fig. 3).

### Functional connectivity in AD

**Figure 4:**
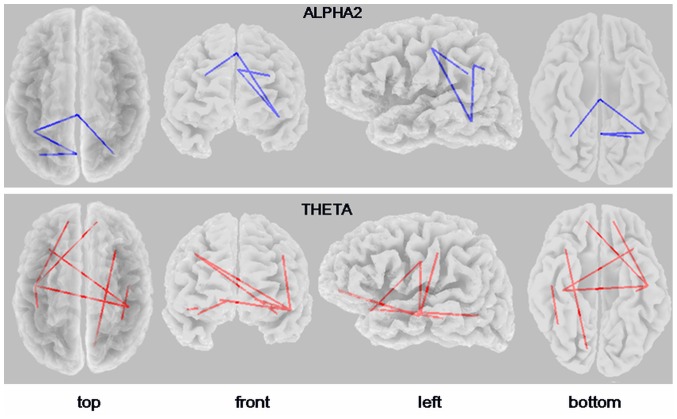
eLORETA wire diagram illustrating cortical areas with significantly decreased (blue wires; threshold: t = −3.49, p<0.05 corrected) and increased (red wires; threshold: t = 4.26, p<0.05 corrected) alpha2 and theta *lagged* phase synchronization, respectively, in patients with Alzheimer's disease vs. controls. Results are displayed on a transparent fiducial cortical surface. The points to which the lines are connected represent the center of the ROIs.

The connectivity pattern of patients with AD, compared with controls, was characterized by reduced alpha2 lagged phase synchronization of the medial frontal and medial parietal region with the temporal and inferior parietal cortex (*t*_max_ = −3.56, p = 0.037 corrected). In addition, there was increased lagged phase synchronization in the theta band, involving mainly temporal and frontal connections (*t*_max_ = 5.06, p = 0.0032 corrected) (Fig. 4). In particular, there was decreased alpha2 connectivity of the midcentral (medial frontal) and midparietal (precuneus) region with the left posterior temporal cortex, and bilaterally with the inferior parietal cortex. Increases in theta connectivity were seen specifically between the left temporal cortex and prefrontal and central areas. Increased theta connectivity also affected right parieto-temporal and fronto-occipital regions as well as interhemispheric connections between the left anterior temporal/inferior frontal cortex and the central cortex. Lagged phase synchronization values of the connections showing significant between-group difference (p<0.05 corrected) in alpha and theta bands are given in Table 3.

**Table 3:** Lagged phase synchronization values of cortical regions showing significant difference in functional connectivity between the patient and control groups.^a^

Bands	Cortical Regions		LPS values		T-value
			AD patients	Controls	
Alpha2	L. Temporal	mid-central	0.091±0.067	0.14±0.090	−3.49
(10–13 Hz)	L. Temporal	mid-parietal	0.096±0.063	0.14±0.077	−3.55
	R. Parietal	mid-central	0.11±0.080	0.15±0.082	−3.56
	R. Parietal	mid-parietal	0.071±0.052	0.10±0.051	−3.52
Theta	L. Temporal	L. Prefrontal	0.074±0.040	0.051±0.027	4.27
(4–8 Hz)	L. Temporal	R. Prefrontal	0.098±0.056	0.063±0.029	5.06
	L. Temporal	L. Central	0.089±0.055	0.060±0.033	4.32
	L. Temporal	R. Central	0.087±0.043	0.067±0.019	4.30
	L.ant. Temporal	R. Central	0.070±0.036	0.050±0.017	4.65
	R. Temporal	R. inf. Parietal	0.076±0.044	0.051±0.030	4.60
	R.ant. Temporal	R. Occipital	0.094±0.055	0.066±0.031	4.39

Data are mean ± SD unless otherwise noted.

a Significant differences corrected for multiple comparisons. AD, Alzheimer's disease; LPS, lagged phase synchronization; ant, anterior; inf, inferior; R, right; L, left.

### Functional connectivity and APOE genotype

**Figure 5:**
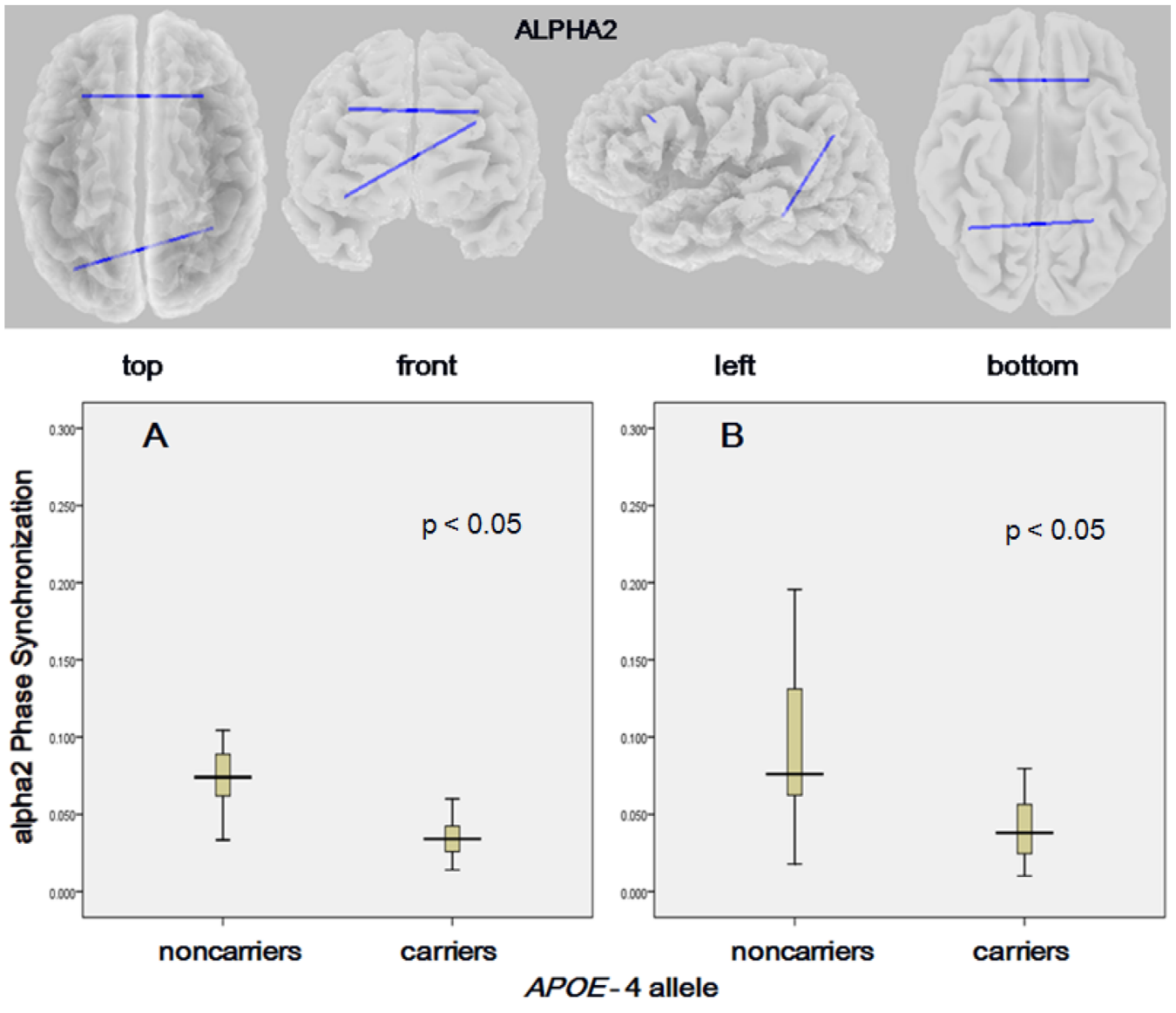
eLORETA wire diagram (top) and boxplots (bottom) of cortical areas with significantly decreased alpha2 *lagged* phase synchronization (blue wires) in *APOE*-4 carriers vs. noncarriers among patients with Alzheimer's disease (threshold: *t* = −3.98; p<0.05, corrected). The significant connectivity wires are displayed on a transparent fiducial cortical surface. The points to which the lines are connected represent the center of the ROIs. The boxes represent interquartile ranges of lagged phase synchronization between A) frontal areas and B) parieto-temporal areas. The lines across the boxes indicate medians, and the whiskers show the highest and lowest values, excluding outliers.

The comparison between *APOE-*4 carriers and noncarriers in the total sample of AD patients showed no significant difference in lagged phase synchronization (*t*_max_ = 2.96; p = 0.81, two-tailed, corrected). We further explored differences in functional connectivity according to *APOE* status in AD subgroups. Because of the small number of patients in the severe AD group (n = 17), data from patients with moderate and severe AD were pooled. The comparison of carriers and noncarriers in the subgroup of moderate/severe AD revealed no statistical significance (*t*_max_ = 2.94; p = 0.88, two-tailed, corrected). However, when analyzing carriers vs. noncarriers among patients with early AD, we found decreased alpha2 lagged phase synchronization between lateral frontal areas (*carriers*: 0.034±0.012, *noncarriers*: 0.084±0.055; t = −4.08, p = 0.045 corrected) and parieto-temporal areas (*carriers*: 0.040±0.019, *noncarriers*: 0.10±0.067; t = −3.98, p = 0.049 corrected) across hemispheres (Fig. 5).

### Correlation of functional connectivity with cognitive scores

**Figure 6:**
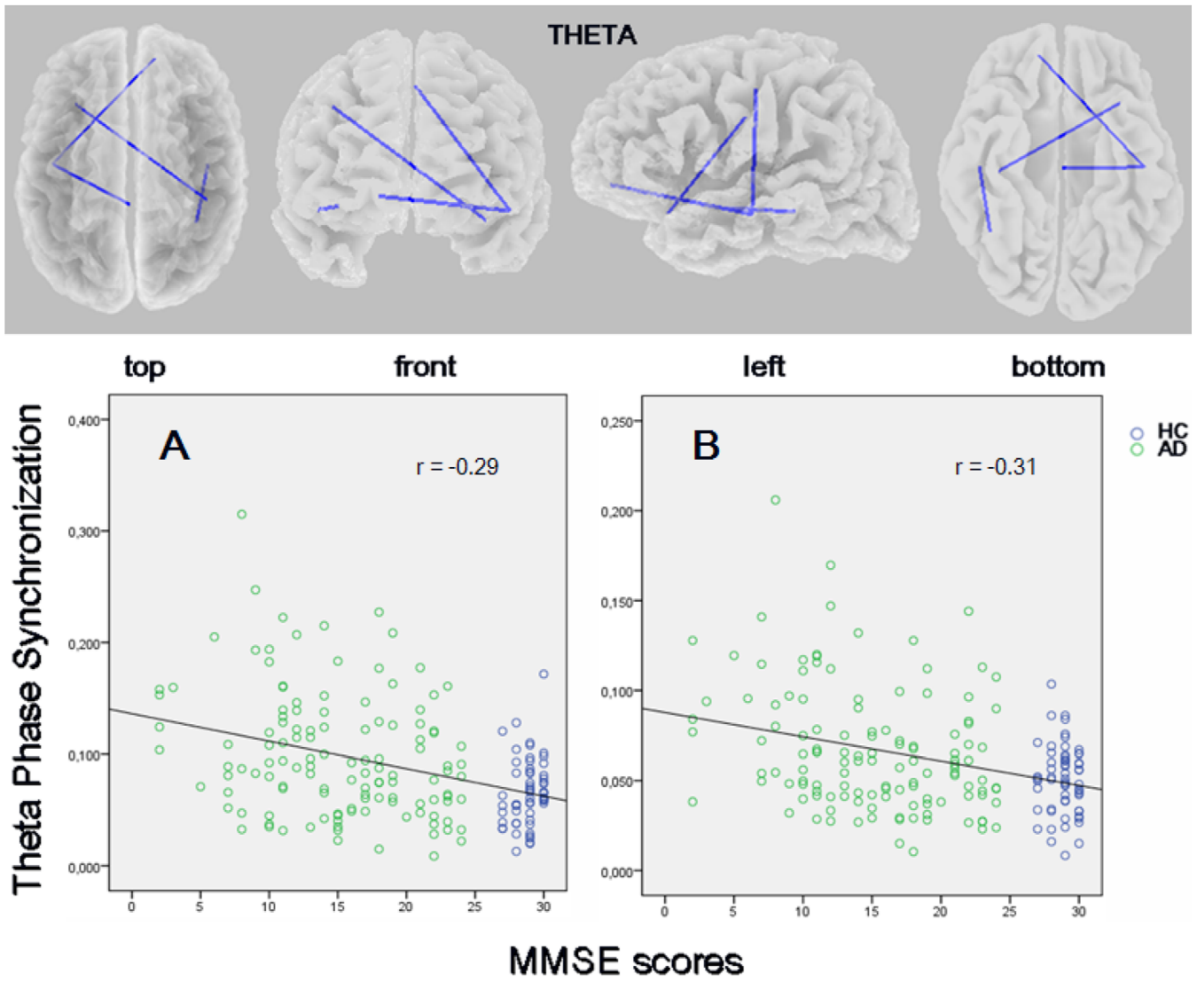
eLORETA wire diagram of significant correlations between theta *lagged* phase synchronization values and the Mini-Mental State Examination scores in patients with Alzheimer's disease and controls (threshold: *r* = −0.27; p<0.05, corrected). The blue color of the wires indicate a negative correlation. Results are displayed on a transparent fiducial cortical surface. The points to which the lines are connected represent the center of the ROIs. The bottom panel shows scatterplots of the strongest correlations. The results displayed correspond to the connectivity between A) left temporal-right prefrontal cortex, and B) left anterior temporal-right central cortex.

The eLORETA correlation analysis indicated that lagged phase synchronization in the theta band had a negative correlation with the MMSE scores: the higher the functional connectivity in the (slow) theta band across these regions the more the cognitive decline (lower MMSE scores). This included those cortical regions showing the highest between-group difference in theta lagged phase synchronization, namely the left temporal-right prefrontal (*r* = −0.31; p = 0.019, corrected) and left anterior temporal-right central (*r* = −0.29; p = 0.022, corrected) cortex. Significant correlations of theta connectivity with the MMSE scores were also observed between the left temporal-midcentral area and the right temporal-inferior parietal cortex (*r* = 0.28 corrected) (Fig. 6).

## Discussion

We used a measure of physiological nonlinear connectivity, namely lagged phase synchronization, to determine differences in resting-state functional connectivity in patients with AD compared with elderly controls, and to assess the effects of *APOE* genotype on this activity. Abnormalities in cortical oscillations were also explored. Patients with AD exhibited a significant decrease in alpha1 oscillations in parieto-occipital regions compared with controls, and those carrying the *APOE*-4 allele had reduced alpha1 activity in the left inferior parietal and temporo-occipital cortex relative to noncarriers. The patients showed a pattern of decreased alpha2 lagged phase synchronization between the medial frontal/parietal region and the left temporal and the bilateral inferior parietal cortex, along with increased connectivity in the theta band in different cortical regions, where temporal connections were particularly compromised. Some of these functional connections in the theta band correlated negatively with the MMSE scores. Functional network disruption in patients with early AD carrying the *APOE*-4 allele was characterized by decreased interhemispheric alpha2 connectivity between frontal and parieto-temporal areas compared with noncarriers. There were no *APOE*-4-related connectivity abnormalities in the subgroup of patients with moderate/severe AD.

### Abnormal alpha oscillations and *APOE* genotype

The abnormalities found in alpha oscillations in the parieto-occipital regions in patients with AD were more prominent over the medial parietal cortex, particularly in the precuneus, while the reduced alpha oscillations in patients carrying the *APOE*-4 allele compared with noncarriers had higher maxima in the inferior parietal cortex. Of note, these two regions of the parietal lobe are part of the DMN, a brain circuit typically active during rest [37]. The alpha rhythm or enhanced alpha activity in posterior regions is one of the most prominent electromagnetic changes in the human brain, representing a distinctive feature of the normal brain in the waking resting-state. Abnormalities in alpha power or its typical parieto-occipital distribution may sometimes represent an early or the only sign of cerebral dysfunction in neuropsychiatric disorders [61]–[64]. In addition, it is well established that patients with AD present with clinical symptoms of temporal (e.g., memory impairment) and parieto-occipital deficits (e.g., visuospatial impairment) even at early stages of the disease [1], [13]. Therefore, the reduction in alpha power found in patients with AD in this study, along with a tendency towards an increase in slow oscillatory activity most likely represents disease- and genotype-related resting-state regional dysfunction.

These eLORETA source-localization results are consistent with those of neurophysiological studies suggesting that reduced alpha oscillations in temporal, limbic and parieto-occipital regions, as well as increased slow activity sources in AD are associated with the global cognitive level [65], [66] or the presence of *APOE-*4 allele [33]. They are also in line with findings from PET and SPECT investigations demonstrating pronounced hypometabolism or reduced blood flow in posterior brain regions in AD, especially in the parietal cortex [67], [68]. Together, these data support the notion of temporal and parieto-occipital cortical deficits, and resting-state DMN dysfunction as important neurobiological features of AD, and confirm a link between *APOE*-4 and cortical function phenotype.

### Aberrant patterns of alpha and theta connectivity

Our connectivity analyses revealed the presence of both decreases and increases in lagged phase synchronization, as a measure of functional connectivity, affecting alpha and theta frequency band, respectively. Consistent with our findings of decreased alpha2 connectivity in left temporal and medial/lateral parietal regions, previous neurophysiological studies using coherence or synchronization likelihood noted that synchronized activity, particularly in the alpha band, disintegrates in AD and other types of degenerative dementia, indicating disruption in neuronal comunication [12], [17], [19], [38], [39]. Furthermore, fMRI age-related connectivity loss is often found in the temporal lobe as well as in posterior areas of the DMN, including the posterior cingulate, medial parietal (i.e., precuneus) and inferior parietal cortex, which are selectively vulnerable to early amyloid deposition or show decreased metabolism early in the course of AD [14], [37], [69], [70].

Taking into account the neurobiological basis of AD (e.g., axonal disruption, synaptic loss and cholinergic deficits) and its implications for the disconnection hypothesis in this disorder, increased connectivity in patients with AD has been seen as a paradoxical phenomenon in several fMRI studies, based on BOLD signal changes as an indirect measure of neural activity [15], [16], [36], [70], [71]. Increases in neural synchronization in AD have been reported in a few resting-state functional connectivity studies using EEG or MEG techniques, which measure neural activity (i.e., postsynaptic potentials) directly and noninvasively [18], [20], [22]. For instance, an MEG report on AD connectivity by Stam et al. using synchronization likelihood indicated a loss of long-distance alpha interactions with concomitant increase in parietal lobe connectivity [22]. More recently, another resting-state MEG report suggested both decreases and increases in functional couplings in different frequency bands, as indicated by logistic regressions on linear and nonlinear connectivity values. However, the topographic distribution of these connectivity deficits was not explored [20]. Sankari et al. using a comprehensive EEG coherence analysis found decreased temporo-parieto-central functional connections along with a hyperconnectivity pattern in left temporo-parietal and frontal regions [18]. Interestingly, although different frequency bands were involved in the aforementioned studies reporting regions with functional hyperconnectivity in AD, this activity has consistently been found most pronounced in the theta band, which is in line with the increase in theta phase synchronization observed in patients with AD in our study.

Most authors proposed that increased resting-state functional connectivity in AD might reflect a compensatory mechanism in an attempt to counteract the inefficiency of memory networks and make up for the decrease in cognitive functions caused by the disease [36], [69], [71]. This argument is mainly based on evidence of increased connectivity in resting fMRI, particularly in prefrontal networks and in anterior/medial temporal regions, which are sites of early pathological changes in AD. Reports of hyperconnectivity in MEG recordings of patients with amnestic mild cognitive impairment, a disorder considered as a prodromal stage of AD, provides support to this view [72], [73]. Our findings and evidence of enhanced theta activity in association with cognitive-task induced activation in humans [74], and with recovery of memory function [75] in rats further suggest that increased phase synchronization in theta band may represent the underlying neural activity of a compensatory network in AD.

An alternative explanation of the compensatory mechanism may be that damage to a specific network enhances connectivity within regions that normally feature an anticorrelated relation to the damaged network [12]. However, since theta connectivity correlated with the MMSE scores, suggesting a worsening of increased activity with the progression of memory decline, the possibility that higher connectivity in a slow band (i.e., theta band) relates to pathology or dysfunctionality (e.g., excitotoxicity or neuronal failure) rather than to compensation cannot be ruled out. EEG reports showing a negative correlation between slow wave global synchronization and AD severity provide support to this view [76], [77]. Altogether, our findings and those of recent EEG/MEG studies indicate that increased theta synchronization between certain brain regions in AD does not represent a paradoxical phenomenon, suggesting a role for increases in functional connectivity in AD pathophysiology.

### *APOE*-4 effects on AD functional connectivity

Despite an increasing body of literature on *APOE*-brain networks relationship in cognitively healthy individuals, little has been reported on the effect of *APOE* genotype on resting-state functional connectivity in AD. Our results showed a significant decrease in lagged phase synchronization in the alpha2 band affecting lateral frontal and parieto-temporal areas across hemispheres. An EEG study by Jelic et al. using classical coherence analysis compared 10 patients carrying two ε4 alleles, 14 with one ε4 allele and 17 noncarriers with 18 healthy subjects in whom the *APOE* genotype was not determined. As a result, homozygous patients showed reduced connectivity with temporal and parietal regions [35]. However, the fact that a direct comparison of AD patient subgroups according to *APOE*-4 status was not performed, and additionally, the control group had unknown *APOE* genotype, limits the interpretation of the findings regarding the role of *APOE-*4 on AD functional connectivity. Kramer et al. in an EEG synchronization likelihood study assessed *APOE*-4 effects on functional connectivity across patients with AD and subjects with subjective memory complaints who served as controls. They noted that among *APOE*-4 carriers, the patients had decreased alpha connectivity compared with controls, although synchronization likelihood values were significantly higher in carriers vs. noncarriers in both groups. This led to the suggestion that the effects of AD and *APOE*-4 on global functional connectivity are independent and opposite [21]. The findings of our work cannot be directly compared with those of Kramer et al. [21] as they used averaged scores of synchronization likelihood values of all electrodes for each frequency band to measure global connectivity. Therefore, the topographic distribution of the compromised networks could not be assessed. Nevertheless, the two studies identified abnormalities in alpha band as neural activity underlying *APOE*-4-related connectivity deficits in AD.

Evidence from a recent MRI study by Filippini et al. provides support to our findings of decreased connectivity in prefrontal regions in AD patients carrying the ε4 allele. They looked at regional atrophy and transcallosal connections in cognitively normal *APOE*-4 carriers, and demonstrated that *APOE*-4 polymorphism was associated with aging-related volume loss in the prefrontal callosal tracts [78]. A study by Brown et al. found that, compared with noncarriers, elderly subjects carrying the *APOE*-4 allele exhibited pronounced age-related connectivity decreases along with loss of regional cortical thickness in the lateral parietal cortex and other brain regions [29]. It is therefore conceivable that these anatomical connectivity deficits may be more accentuated in pathological aging, leading to the frontal and parietal network disruption observed among AD patients carrying the *APOE*-4 allele in this study. Taken together, our results and those of previous genetic studies in healthy young and elderly subjects, suggest that *APOE*-4, as a risk factor for AD, is associated with changes in the intrinsic functional organization of brain networks in cognitively normal carriers [31], [32], [78], [79], even in the absence of amyloid plaques [80], and with some degree of frontal and parietal disconnection at the stage of early AD. Patients with long-term and severe dementia, however, might show functional connectivity patterns determined by the cognitive level or disease-specific factors rather than by the *APOE* susceptibility gene.

Some study limitations are acknowledged, including the small number of electrodes used for EEG recording and data analyses that may affect the source localization results. However, the good localization property of the LORETA tomography has been evidenced in several studies applying 19-channel EEG systems. These studies have successfully localized functional abnormalities in cognitive disorders [33], [65] and correlated LORETA three-dimensional current source density with anatomical modules of synaptic activity measured by diffusion spectral imaging [81]. Since our analyses focused on functional connectivity in the 1–30 Hz frequencies, we cannot rule out that *APOE*-related lagged phase synchronization abnormalities during rest may appear within certain range of the gamma band. Further neurophysiological studies using high-channel EEG/MEG systems may shed some light on the role of resting-state gamma connectivity in dementia. Another potential limitation to be considered is that an *APOE*-4 homozygous group of AD patients was not included, due to the small number of ε4 carriers with two alleles (n = 5). Therefore, functional connectivity abnormalities possibly related to the dose of the *APOE*-4 allele (homozygous vs. heterozygous) could not be explored.

## Conclusion

Overall findings indicate that in addition to regional cortical dysfunction, there is impairment in resting-state functional connectivity in alpha and theta bands in AD. This activity affects various brain networks, where temporal lobe (i.e., fronto-temporal and parieto-temporal) connections are particularly compromised, and theta hyperconnectivity likely representing a compensatory mechanism. Although *APOE*-4 is associated with increased vulnerability to AD, as a genetic risk factor, it appears to have a negative impact on cortical rhythms and functional brain networks even after the development of the disease that is particularly manifested during the early stage of the disorder. The altered connectivity pattern in later stages might be determined by cognitive factors or disease-specific abnormalities. These findings warrant further investigation and suggest that the patterns of functional network disruption, as indicated by lagged phase synchronization measures, may potentially represent neurophysiological or phenotypic markers of AD, and aid in early detection of the disease.
